# Neuroinflammation in Ischemic Stroke: Mechanisms, Systemic Interactions, and Therapeutic Targets

**DOI:** 10.1007/s12975-026-01442-9

**Published:** 2026-05-26

**Authors:** Eduardo Soares, Willeke F. Westendorp, Jonathan M. Coutinho, Matthijs C. Brouwer, Diederik van de Beek

**Affiliations:** Department of Neurology, Amsterdam UMC, University of Amsterdam, Amsterdam Neuroscience, PO Box 22660, Amsterdam, 1100 DD The Netherlands

## Abstract

Ischemic stroke is the second leading cause of death worldwide and its global burden is predicted to increase over the upcoming decades. Acute therapies primarily focus on recanalization of the occluded blood vessels by intravenous thrombolysis and endovascular thrombectomy, but these therapies are not suitable for all patients. Also, in case of successful reperfusion, there remains a risk of secondary tissue damage known as ischemia-reperfusion injury. Inflammation plays a crucial role in both the infarcted tissue and ischemia-reperfusion injury after stroke. Innate and adaptive immune systems may drive an exacerbated inflammatory response after stroke, impacting patients’ outcome. Research in stroke pathophysiology and the role of the immune system after stroke suggests that outcome may be improved by using immune-based therapies preventing this secondary tissue damage. Here we display a comprehensive description of chronological immune events at the cellular and molecular levels in different compartments after stroke and discuss the potential neuroprotective role of regulatory T cells (T_regs_). We connect both anti and pro-inflammatory cascades in parallel within different body compartments over time and explore the potential for manipulation of specific immune events to develop new therapies.

## Introduction

Stroke is a major health concern, ranking among the top three causes of morbidity and mortality worldwide [1]. Approximately 80% of strokes are ischemic, resulting from arterial thrombus formation or chronic thickening of small blood vessels, both leading to cerebral blood vessel occlusion and resulting ischemia of brain tissue [2, 3]. The prognosis of patients with ischemic stroke is poor, as 15% of patients die and 40% of survivors are disabled [4]. Acute therapies for ischemic stroke focus on re-establishing blood flow to the affected brain tissue and consist of intravenous thrombolysis and endovascular thrombectomy. These treatments have improved the prognosis of ischemic stroke considerably, but the outcome has to be further improved.

Insights about stroke pathophysiology and the immune system suggest that outcome after stroke may be improved with immune-based therapies that target secondary tissue damage. The tissue damage caused by the ischemic event triggers a profound inflammatory response that negatively impacts the prognosis of ischemic stroke patients. However, due to the complexity of the pathophysiology of ischemic stroke, involving the disruption of several biological processes, the precise molecular and cellular mechanisms influencing patients’ outcomes remain largely unknown. Research has focused on the acute immune response occurring in peripheral blood of patients after stroke in the hours to days after stroke onset. The inflammatory response following an ischemic stroke exhibits substantial temporal variations and can differ across multiple compartments that exhibit pronounced temporal heterogeneity, including the blood, lymph nodes, and the brain adjacent areas. As a result, the temporal knowledge of the cellular and molecular mechanisms that drive neurological deterioration in face of the newly described tissue repair remain relatively disconnected.

Much of our mechanistic insight into inaccessible experimental human tissues come from experimental models of cerebral ischemia, which often lack recanalization and therefore primarily capture immune mechanisms of sustained ischemia, rather than those occurring after successful reperfusion in patients. Based on those models, we discuss emerging concepts that indicate that anti-inflammatory components of the immune system have the potential to mitigate the deleterious effects caused by inflammation and induce tissue regeneration in specific situations and time frames [5–8].

To bridge several outstanding gaps that are not yet clearly connected, this review provides an overview of current understanding of the immune response after ischemic stroke, delving in cellular as well as molecular aspects in a temporal manner. Recent literature was selected from experimental and clinical studies addressing temporal immune responses across multiple tissue compartments after ischemic stroke, with emphasis on studies reporting time-resolved immune changes. We adopted an immunological phase definition, as defined by Tsai and colleagues [9]. Based on differential mass cytometry (CyTOF) data from peripheral blood of ischemic stroke patients compared to control subjects they defined three distinct inflammatory phases: (a) an early phase or acute immune response after two days, (b) an intermediate phase after five days, and (c) a late phase after ninety days of stroke onset [9]. We integrate these findings across the central nervous system (CNS), peripheral immune system, and lymphoid tissues, with a specific focus on the interplay between pro and anti-inflammatory responses. We will provide insights in emerging neurological fields, such as the glymphatic system and gut-brain axis, and their potential role in ischemic stroke. Finally, an assessment of potential new treatment strategy will be provided, along with a survey of potential research directions that hold promise for advancing the field. By compiling and integrating these temporal and multi-compartment insights, this review aims to provide a framework to guide the design of targeted immune-based therapeutic strategies after ischemic stroke.

### Brain Response to Ischemia

A substantial proportion of ischemic stroke survivors have focal neurological deficits, such as hemiparesis and aphasia, or may face cognitive decline [10, 11]. Tissue injury may be a result of the ischemia itself [12], injury due to reperfusion [13, 14], or arise as secondary effects of immune mediated inflammation [15].

Traditionally considered an immune-privileged site, the CNS is accepted to be an immune-specialized organ. The maintenance of tightly regulated crosstalk with the peripheral immune system is maintained by microglial surveillance and meningeal lymphatic drainage pathways [16–18]. Ischemic stroke destabilizes this tightly regulated system by damaging the neurovascular unit, promoting blood–brain barrier (BBB) breakdown, and allowing CNS antigens and inflammatory mediators to access the systemic immune system [19–21] as discussed later in this review. This shift from immune-controlled surveillance to uncontrolled neuroinflammation results in quick recruitment of peripheral immune cells into the brain, amplifying secondary injury and worsening outcomes [22–25].

**Fig. 1:**
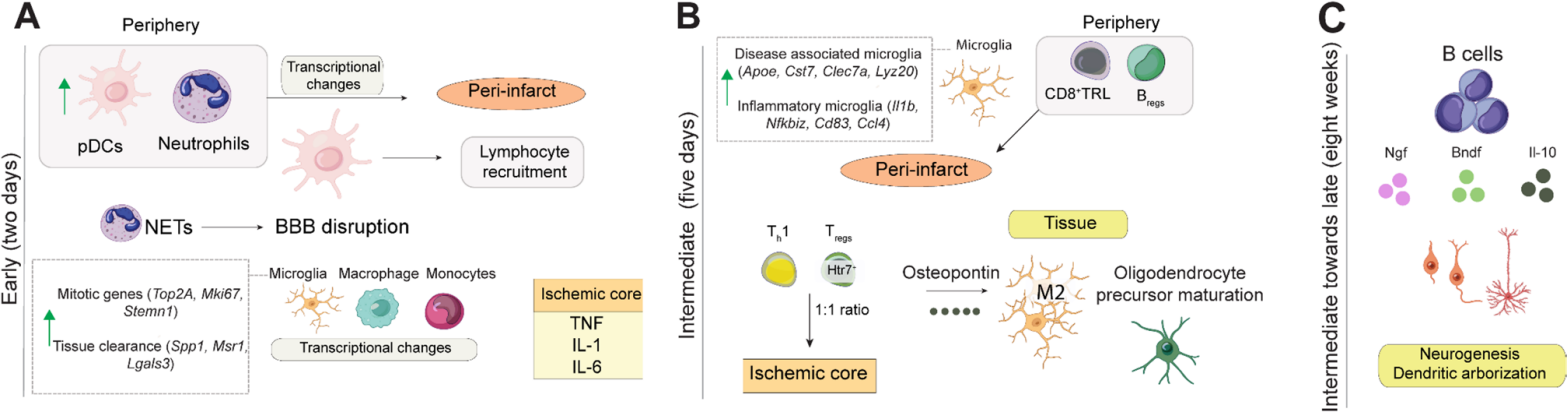
Chronological immune cascades in the ischemic brain (**A**) early response involves events described until 72 hours after induction of stroke in experimental models. These models point out increased levels of tumor necrosis factor (TNF), interleukin (IL)-1 and IL-6 in the ischemic core concomitant with neutrophil recruitment followed by BBB disruption. At the same time, the resident microglia, monocytes and macrophages undergo transcriptional changes and recruitment of plasmacytoid dendritic cells (pDCs) is observed in the border regions of the ischemic core, as early as 24 hours and participate in the recruitment of other immune components to the brain such as T cell lymphocytes, including T_regs_ (**B**) intermediate response includes significant immune population changes described after 72 hours. During this phase, transcriptional changes in the resident microglia are observed compared to the early phase. This phase is characterized by the enrichment of components of the adaptive immune system, mainly T cells. pDCs recruited T cells migrate from the peri-infarct area to the ischemic core. In this stage, a 1:1 ratio of Th1 and T_regs_ is observed. Htr+ T_regs_ will promote microglia polarization toward the M2 phenotype and tissue repair via oligodendrocyte precursor maturation. At different stages, recruitment of lymphocytes with regulatory properties is observed, such as T_regs_, CD8+ T regulatory-like cells (CD8+ TRLs), and regulatory B cells (B_regs_). Although some of this recruitment can be observed in very early stages, neuroprotective roles are only detected from the intermediate phase onwards (**C**) Late immune response is characterized by B cell activity in remote brain areas promoting recovery events such as dendritic arborization. Peripheral events are indicated in the grey boxes

Studies involving animal models and human autopsies have revealed an immune imbalance triggered by ischemic stroke in the brain. In the immediate aftermath after stroke, signaling molecules, such as brain derived antigens, damage-associated molecular patterns (DAMPs), cytokines and chemokines [26], trigger the activation of pro-inflammatory cells. This activation leads to a prompt systemic inflammatory response through the release of pro-inflammatory cytokines such as tumor necrosis factor (TNF), interleukin (IL)−1 and IL-6 among other signaling molecules, resulting in blood-brain-barrier (BBB) disruption (Fig. 1a) [27, 28]. This neuroimmune activation is now considered a central determinant of stroke outcome [29]. Nevertheless, the understanding of the interplay between resident brain cells and the migrating peripheral immune components in the post-ischemic brain, along with their contribution to tissue damage and repair, remains poorly understood [30, 31].

#### Early Immune Response or Acute Phase

Neutrophils play an important role as immediate responders in ischemic brain conditions, being the first blood-derived cells to infiltrate the affected tissue [32]. Histological examinations of patients who died of ischemic stroke reveal that neutrophils are recruited from the periphery to the brain within 24 h of cerebral ischemia [33]. Neutrophil enrichment in the infarction core is linked to very early stroke-associated disturbances of normal physiology, including disruption of the BBB and cerebral edema in addition to the brain injury of the stroke itself [34] (Fig. 1a). Their activation drives the release of inflammatory mediators and the release of intravascular and intraparenchymal neutrophil extracellular traps (NETs) in the ischemic brain, inducing thrombosis and amplifying inflammation [35]. Elevated NET deposition correlates with impaired vascular remodeling and poorer neurological outcomes, and experimental neutrophil depletion reduces BBB breakdown and enhances neovascularization [35].

In parallel to the infiltration of peripheral immune cells, resident glial cells play a central role in shaping the neuroinflammatory response following ischemic injury. In ischemic stroke, microglia rapidly activate as early effectors of neuroinflammation, mediating cytokine production, phagocytosis, and recruitment of peripheral immune cells, whereas reactive astrocytes regulate inflammatory signaling and BBB integrity during both injury and repair [29, 36–40]. Reperfusion further amplifies these processes through oxidative stress, endothelial activation, and increased leukocyte recruitment, thereby contributing to secondary inflammatory injury in the ischemic brain [26, 36].

Recent single-cell transcriptomics on brain and blood after experimental stroke revealed strong phenotypic differences in immune components following cerebral ischemia, such as brain-associated macrophages as quickly as two days post-stroke [15]. Whereas other components, such as microglia, monocyte-derived macrophages and neutrophils, evolving toward chronic neuroinflammatory-like programs by 14 days. Other components, such as microglia, monocyte-derived macrophages and neutrophils, also show early changes at day 2, accumulating over the time course of 14 days. For example, microglial transcriptomes showed strong enrichment of proliferation-associated genes at day two as a possible response to the pronounced cellular loss. At the same time point, another microglia subpopulation with a phagocytic signature has been identified based on transcriptomic profiles. The early (day 2) phenotype is divergent from the intermediate (day 14) one, which resembles molecular signatures associated with broader patterns of neuroinflammation, such as in Alzheimer’s disease [41]. Infiltrating leukocytes acquire distinct transcriptional signatures compared to their circulating counterparts, indicating that the ischemic microenvironment dictates immune cell fate and function [15].

Dendritic cells (DC), specifically plasmacytoid dendritic cells (pDC), are quickly enriched in the periphery after stroke [9]. This peripheral migration and DC infiltration peaks in the infarction core 72 h after stroke [42]. Brain dendritic cells (bDC) are recruited mainly in the border regions of the infarcted area, participating in the recruitment of other immune components to the brain, such as T-cell lymphocytes [42]. T-cell lymphocytes, in turn, are important dual-role cellular players typical of the intermediate immune response (Fig. 1a) [9].

#### Intermediate or Subacute Immune Response

After the initial influx of innate immune cells around the infarction site, various T-cell subsets progressively become prominent in the ischemic brain[8, 9]. Although lymphocyte infiltration in the ischemic brain is a long-lasting process [43], accumulated evidence indicates cerebral T cell proliferation rather than constant T cell invasion [44].

T cells initially accumulate in the peri-infarct region and progressively migrate towards the lesion core, where CD4⁺ T cells amplify neuroinflammation by releasing IFN-γ and promoting astrocytic IL-1β, IL-6, and TNF-α production [45]. As inflammation proceeds, T_regs_ expand to counteract this excessive pro-inflammatory cytokine signaling, in a process supported by IL-6 and TNF-α-driven T_reg_ proliferation [46]. CD8⁺ T cells persist in the brain for weeks, adopting delayed transcriptional programs enriched for IL-6 and IFN-γ expression [47], indicating that both CD4⁺ and CD8⁺ subsets contribute to prolonged adaptive immune activity during recovery (Fig. 1b).

In an experimental stroke model, massive accumulation of T_regs_ has been reported 14 days after disease onset shifting from their typically low abundance in circulation toward near parity with effector Th1 cells within the lesion [5]. Analysis of T_regs_ infiltration after chemical egress inhibition of activated T cells from lymph nodes indicates that brain infiltrated T_regs_ are amplified in the cervical lymph nodes a few days before brain infiltration, with further expansion in the brain thereafter. According to Shi et al. (2021), once in the brain, T_regs_ may limit secondary infarct growth by suppressing pro-inflammatory cytokine production and repressing innate and adaptive immune activation. A deeper look into the mechanism of T_regs_-mediated neuroprotection in the post-stroke brain suggests that amphiregulin secreted by T_regs_ will act as important inhibitors of neurotoxic astrogliosis [5, 9] (Fig. 1b). These results align with previous results showing that T_regs_ cells prevent secondary infarct growth by counteracting excessive production of pro-inflammatory cytokines and by modulating invasion and/or activation of lymphocytes and microglia in the ischemic brain [7].

Further evidence on T_regs_-mediated responses during stroke shows that the interplay between T_regs_ and microglia creates a microenvironment enriched with osteopontin [8]. The osteopontin augmentation enhances microglia polarization toward an anti-inflammatory and reparative phenotype that will lead to tissue regeneration, such as white matter repair at the chronic stages of ischemic stroke [8]. It has been proposed that boosting T_reg_ numbers may improve long-term outcomes after stroke (Fig. 1b).

Remarkably, T cells have conflicting roles in post-stroke inflammation, likely due to their functional complexity through different pro- and anti-inflammatory functions. While experimental studies suggest that reducing overall T-cell infiltration into the brain may be beneficial [44, 48], specific T-cell lymphocyte populations, such as T_regs_, have been shown to diminish neuroinflammation [5, 7]. Notably, some studies report early Treg activity to be detrimental [49, 50] as T_regs_ may impede beneficial immune clearance or exacerbate microvascular dysfunction under certain conditions. This highlights the importance of timing, local immune context, and whether reperfusion has occurred, in determining whether T_regs_ act as neuroprotective regulators or contribute to secondary microvascular injury. Therefore, future immunomodulatory strategies must consider not only the temporal profile of T_reg_ activity but also the reperfusion status of the affected tissue.

#### Intermediate Towards Late Immune Response (8 weeks)

In contrast to T cells, B-cell lymphocytes appear to infiltrate the brain later in the onset [6, 51, 52] It has been shown, that CD4^+^ T cells play a role in delaying B-cell infiltration into the brain of a murine ischemic stroke model [43]. Brain-infiltrating B-cell lymphocytes seem to ameliorate long-term neuropathology and support tissue regeneration in mice after induced ischemia [6]. Whole brain image analysis has shown that B cells exhibit migration outside the ischemic injury sites between 2 and 8 weeks after ischemia. In these sites, B cells mediate bilateral diapedesis, or leukocyte extravasation, into the parenchyma. B-cell depletion in tMCAO mouse model is correlated with impaired spatial memory, delayed motor recovery and increased anxiety through eight weeks after experimental stroke onset [6]. B-cell lymphocytes are found to produce neurotrophins (e.g., brain-derived neurotrophic factor (BDNF) and nerve growth factor (NGF), in addition to IL-10), supporting plasticity and neurogenesis. In vitro data also confirm a central neurotrophic role of B cells on the survival and homeostasis of neurons, as well as dendritic arborization, in mixed cortical cultures [6]. However, it is important to highlight that the role of B cells after ischemic stroke remains controversial and likely depends on timing, B-cell subtype, and possibly comorbidities (age, sex, vascular risk, reperfusion status). These findings suggest the translational potential of B and T-cell lymphocytes, emphasizing the need for deeper exploration on counterbalancing molecular mechanisms mediated by these cells (Fig. 1c). Similarly to T cells, B-cell phenotype is intrinsically linked to function following stroke — beneficial or detrimental depending on subset, timing, and microenvironment [53].

### Glymphatic System

**Fig. 2:**
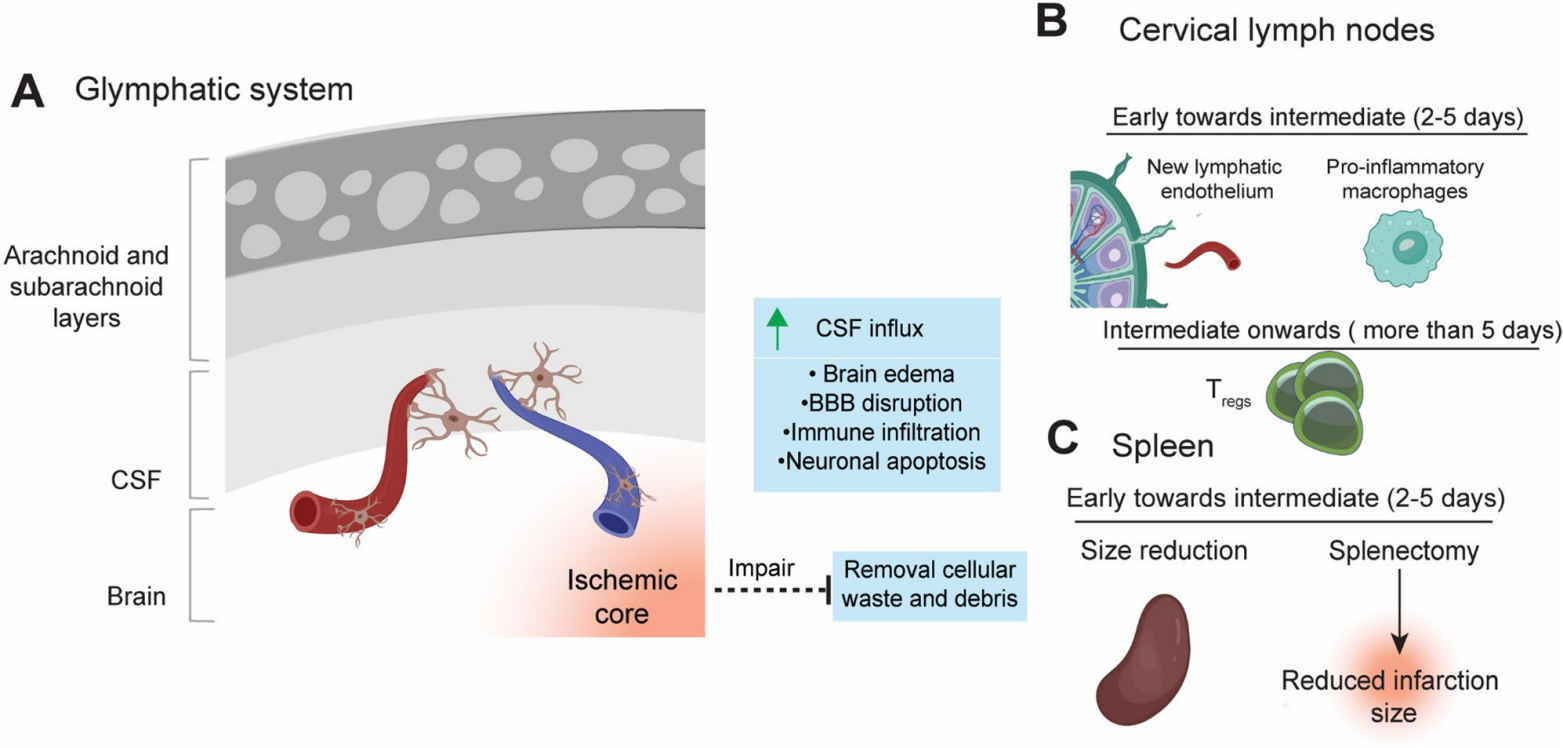
Glymphatic system and secondary lymphoid tissues response after stroke (**A**) In stroke settings, the physiological function of the glymphatic system is impaired and the homeostatic removal of cellular waste and debris will be compromised, leading to tissue-level disturbances. In stroke, CSF influx will increase, but without changes in immune composition. Cerebrospinal fluid (CSF) efflux towards other tissues such as cervical lymph nodes and the proliferation of both pro- and anti-inflammatory immune components (**B**) Events such as proliferation of lymphatic endothelial cells (LECs) and macrophages are observed in the cervical lymph nodes within 24 hours after ischemia. Later on, the cervical lymph nodes are important sites of proliferation for the enrichment of T_regs_ after stroke. (**C**) In the first days after the ischemic insult, a reduction in spleen size is observed in patients and animal models. Splenectomy has been shown to be associated with a reduction in the infarction size in animal models

In recent years, the characterization of the immune components at the CNS borders, such as the glymphatic system has emerged as a compelling area of exploration in the field of neuroinflammation, including ischemic stroke. The glymphatic system has been shown to induce post-injury fluid clearance and, therefore, an essential pathway for the passage of cellular waste and other solutes, such as toxic surplus and cellular debris in the CNS via the cerebrospinal fluid (CSF) [54]. In stroke and neurodegenerative diseases, the glymphatic system’s function is often compromised, leading to disruptions in basic functions such as the clearance of harmful components. Consequently, tissue-level disturbances, such as brain edema, BBB disruption, immune cell infiltration and neuronal apoptosis are observed [55]. Ischemic stroke animal models have indicated increased cerebrospinal fluid influx into the brain after stroke as a driving agent of tissue swelling [56]. However, cellular profiling of CSF after ischemic stroke indicated only a slight increase of immune cells without changes in cellular composition compared to control subjects [57]. Altogether, these facts illustrate that after stroke the draining CSF may play a special outward signaling role in sending extracellular signals from the brain to other sites, such as the cervical lymph nodes (Fig. 2a).

### Cervical Lymph Nodes

Following the pathways in the meninges and CSF, many extracellular solutes are drained to the cervical lymph nodes in health and disease where they might lead to the proliferation of both pro- and anti-inflammatory immune components [16, 58–60]. This CNS-derived antigen efflux rapidly activates immune cells within the nodes and initiates a systemic response after ischemic stroke. The cervical lymph nodes have emerged as pivotal players in the interaction between brain and blood after stroke, especially in the early and intermediate phases. An experimental rat study demonstrated that animals subjected to focal cerebral ischemia, that lymphatic endothelial cells and macrophages start to proliferate in the cervical lymph nodes within 24 h [60]. This proliferation is mediated by upregulation of the prototypical receptor ligand VEGF-C in the cerebrospinal fluid. Co-culturing lymphatic endothelial cells and macrophages revealed that newly formed lymphatic endothelium induced by ischemic stroke stimulates the growth of pro-inflammatory macrophages (Fig. 2b). This surge in pro-inflammatory macrophages exacerbates brain damage after cerebral ischemia, and surgical removal of cervical lymph nodes in mice reduces infarction after cerebral ischemia [60].

However, the cervical lymph nodes are also critical sources of counter balancing immune-regulatory cell efflux during the intermediate to late phases of stroke recovery. While pro-inflammatory macrophages derived from the cervical lymph nodes lead to increased brain damage in the early phase after stroke, the migration of anti-inflammatory T_regs_ from the lymph nodes to the infarction site, at a later stage, will assist in ameliorating the focal damage [5] (Fig. 2b). Ito et al. have shown enrichment of T_regs_ in the cervical lymph nodes after stroke, highlighting in this way that the role of cervical lymph nodes as important niches also for anti-inflammatory or regenerative response. Overall, the limited studies focusing on the role of the cervical lymph nodes after ischemic stroke pinpoint their important role through the proliferation and activation of pro-inflammatory macrophages [60]. Also, cervical lymph node-derived lymphocytes, such as T_regs_, emerge as vital neuroprotective components in the latter phases of ischemic stroke [5]. Notably, T_reg_ contributions remain timing- and context-dependent, and not all studies report uniformly beneficial effects.

Together, these findings position the cervical lymph nodes as a dynamic bidirectional hub coordinating both detrimental and reparative neuroimmune responses after ischemic stroke. In particular, the possibility of cervical lymph nodes-directed T_reg_ stimulation represents a promising translational strategy to selectively enhance neuroprotective immunity while minimizing harmful inflammation could be further tested. Beyond the cervical lymph nodes, other secondary lymphoid organs—most prominently the spleen—also undergo profound and time-dependent remodeling after ischemic stroke, further shaping systemic immune responses and influencing clinical outcomes.

### Spleen

The spleen lacks afferent lymphatic vessels meaning that all cells and antigens enter the spleen via the blood [61]. The communication between the brain and spleen is mediated via the parasympathetic nervous system though both sympathetic and parasympathetic inputs [62]. Numerous studies using animal models have highlighted the spleen's involvement in exacerbating neural injury after ischemic stroke [63–65]. This is supported by evidence that pre-stroke splenectomy decreases ischemic lesion volumes after stroke in mice [63, 64].

Prospective investigation of spleen sizes in patients after ischemic stroke showed spleen contractions on the first days following cerebral ischemia [66] (Fig. 2c). This observation concurs with experimental data showing spleen atrophy four days after ischemic stroke in mice, which is followed by increased splenic levels of CD4 + FoxP3 T_regs_, and may mark an anatomical change between the early and intermediate phases [67]. In addition, there is evidence that splenic innate-like marginal-zone B cells are rapidly lost, impairing early IgM production and increasing susceptibility to bacterial infections after stroke. Adrenergic-mediated loss of splenic marginal zone B cells contributes to post-stroke infection susceptibility [68]. This systemic B-cell disruption may partially explain why some patients develop post-stroke infections, which themselves worsen neurological outcome. Experimental animal model studies have shown that the spleen releases leukocytes that mediate secondary brain injury in the early onset phase [67, 69, 70].

### Bone Marrow

The domains within the bone marrow microenvironment offer niches for the proliferation and differentiation of hematopoietic stem cells and immune cells during inflammation induced by ischemic stroke. The capture of signals from growth factors and cytokines, or components of the extracellular matrix, will regulate specific cellular programs [71]. In the context of ischemic stroke, sympathetic nervous signaling modulates hematopoietic regulation in the bone marrow by activating hematopoietic stem cells [72].

**Fig. 3:**
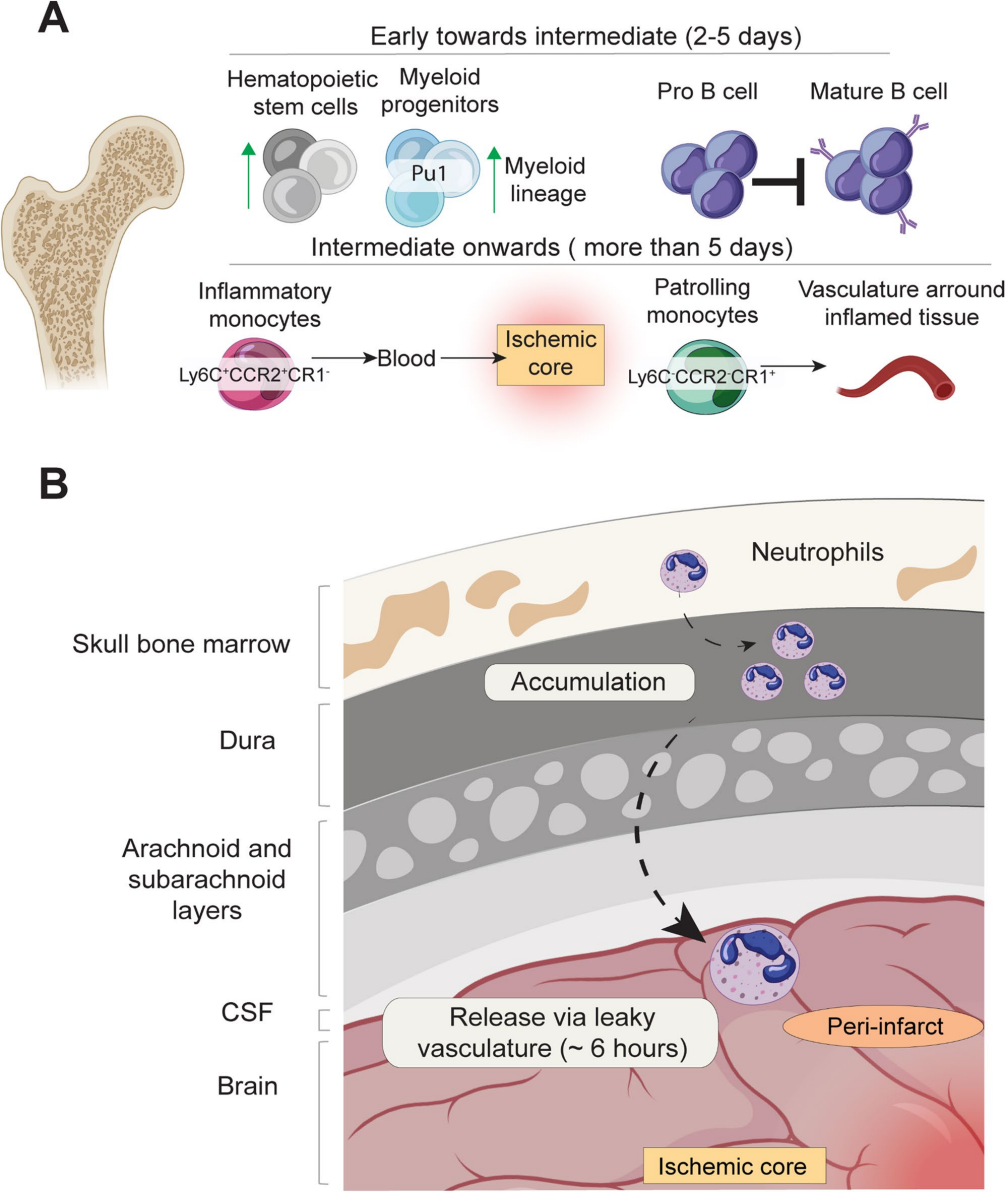
CNS-associated (skull) bone marrow immune response (**A**) sympathetic nervous signalling leads to the activation of hematopoietic stem cells (HSC) in the bone marrow and increased proliferation of PU.1 high myeloid progenitors, which will commit towards the myeloid lineage. At the same time, progenitor B cells are arrested at the beginning of the pro-B cell stage. Besides, inflammatory monocytes (Ly6C+CCR2+CX3CR1-) ill migrate from bloodstream to the site of inflammation, following a chemokine concentration gradient. Whereas patrolling monocytes (Ly6C−CCR2−CX3CR1+) leave the bone marrow to be recruited to the vasculature of injured/inflamed tissues (**B**) Skull-derived neutrophils migrate against the blood flow into the dura, where they accumulate and reach to the brain through leaky vasculature

Imaging analysis in experimental stroke models using mice reveals rapid and heightened activation of the entire hematopoietic lineage after stroke, with an accelerated proliferation of myeloid progenitors in the bone marrow observed four days after stroke. This expansion corresponds to the upregulation of the transcription factor PU.1 [72], known for inducing myeloid lineage commitment in hematopoietic progenitors and myeloid cells [73]. Bone marrow derived myeloid cell populations will therefore largely expand from bone marrow progenitors exhibiting peak cycling activity four days after stroke [72]. Concurrently, the cellular frequencies of lymphocyte precursors in the bone marrow decrease during these early stages [72]. The hypothalamic-pituitary-adrenal axis mediates B lymphopoiesis impairments after stroke in mice. Insights in the brain-bone marrow communication via hormonal long-range signals reveal a stroke-induced arrest in B-cell development, particularly at the pro-B-cell stage [74] (Fig. 3a). These findings contribute to the understanding of the observed relative lymphopenia in the peripheral blood of stroke patients during the initial weeks after stroke.

Bone marrow-derived macrophages play a crucial role in stroke pathology, peaking in the periphery between three and seven days after ischemic stroke. This process is initiated with elevated levels of pro-inflammatory signals (cytokines, MMPs, etc.) attracting bone marrow-derived monocytes into the circulation. Inflammatory monocytes (Ly6C^+^CCR2^+^CX3CR1^−^) migrate from the bone marrow into the blood and ultimately to the site of inflammation, following a chemokine concentration gradient. At the same time, patrolling monocytes (Ly6C^−^CCR2^−^CX3CR1^+^) leave the bone marrow to be recruited to the vasculature of injured/inflamed tissues monocytes [75] (Fig. 3a). The patrolling monocytes reduce inflammatory symptoms by monitoring endothelial integrity [76].

Investigations on bone marrow-mediated immune response after stroke mostly have analyzed cellular percentages in femoral or vertebral bone marrow [72, 74]. In recent years, the discovery of the presence of osseous channels connecting the meningeal tissue with the skull and vertebral bone marrow has added a novel layer of complexity in neuroimmune interactions [77]. Investigations in the field have identified the skull bone marrow as a rapid responder immune reservoir to CNS perturbations [52, 77–79]. For example, in murine models of stroke and aseptic meningitis, neutrophils derived from skull bone marrow are more likely to migrate to the adjacent brain tissue than cells in the tibia as early as 6 h after stroke [78]. These skull-derived leukocytes migrate against the blood flow towards the dura and may reach directly to the brain through the recently induced leaky vasculature [78]. Given this preferential skull neutrophil recruitment observed in the ischemic brain, it may be reasoned that skull-derived neutrophils initiate ischemic neuro-inflammatory cascades [78] (Fig. 3b). Overall, (skull) bone marrow-derived components appear to be relevant in the acute phase of acute stroke, and further exploration is needed to understand their role in later phases after stroke. Although migrating pro-inflammatory immune components to the brain is crucial for tissue clearance after ischemia, temporal targeted inhibition of the above-cited migration pathways may reduce tissue damage caused by excessive inflammation. Similar to the hematopoietic system, outer immune components, such as those derived from the gut are shown to play a crucial role stroke pathophysiology quickly after ischemia.

### The Gut-Brain Axis

**Fig. 4:**
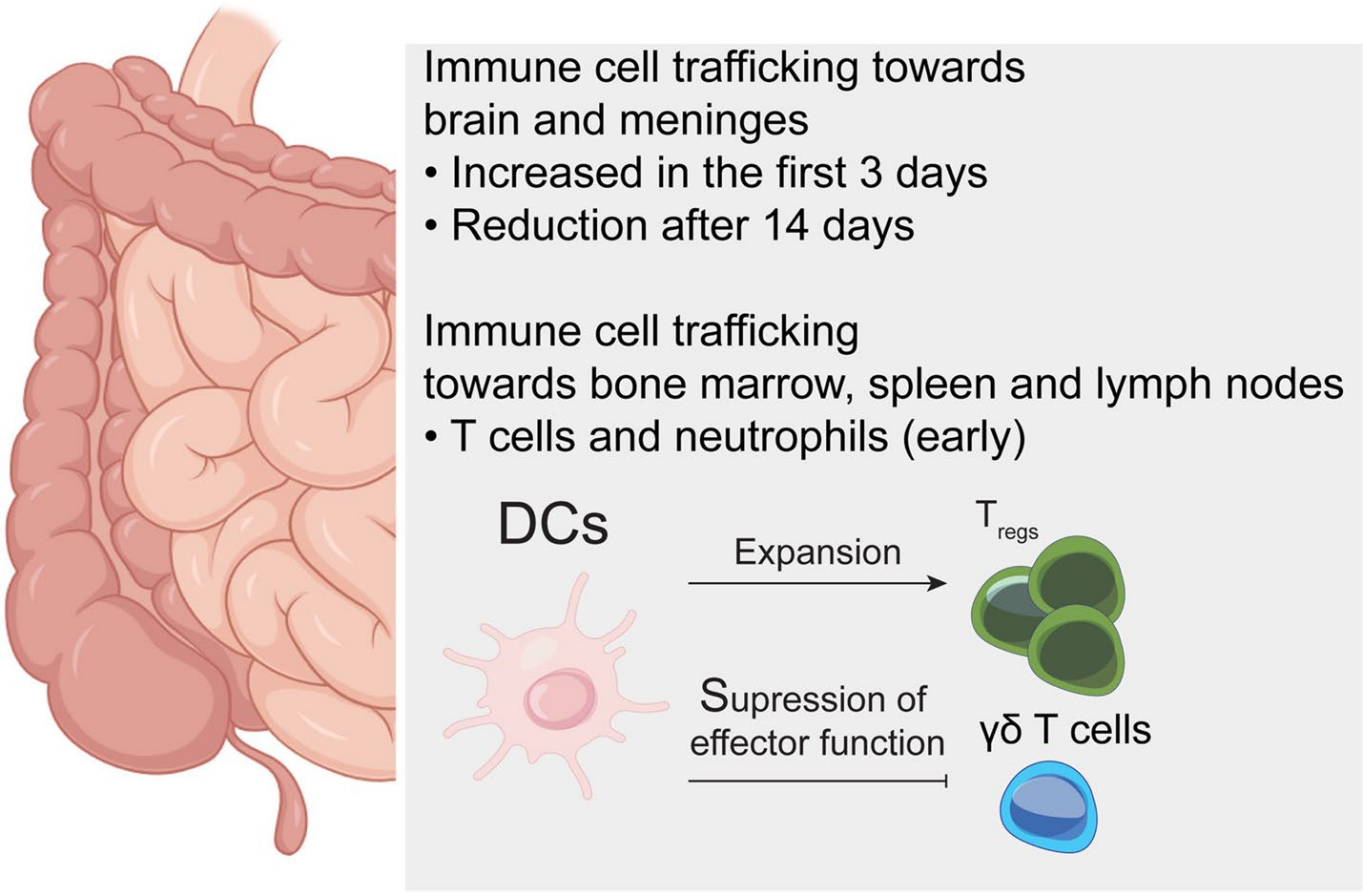
Temporal gut-brain axis mechanisms after ischemic stroke. Immune cells residing in the gut migrate towards the brain and meninges during the early phases of stroke (first three days), and this migration is reduced during the intermediate phase. These gut-derived immune cells, such as T cells and neutrophils, will also migrate towards the bone marrow, spleen and lymph nodes during the early stages. Within the gut microenvironment, dendritic cells (DCs) will induce the expansion of T_regs_ and suppress the effector function of γδ T cells

Bidirectional interactions between the brain and gut are essential in immune-response modulation [80]. As the gut contains over 70% of the immune cells in the body, an ischemic stroke can induce systemic relocation of these immune components, leading to massive migration of these cells to the brain [81, 82]. Using a photoconvertible mouse reporter model subjected to transient occlusion of the middle cerebral artery, one study showed an increased immune cell trafficking from the small intestine to peripheral lymphoid organs and CNS (brain and meninges) in the first three days after stroke in mice which was subsequently reduced during the intermediate phase (14 days) [83]. The number of immune cells that migrated from the small intestine to the brain and meninges in the first three days was even higher than that observed from lymphoid compartments such as bone marrow, cervical lymph nodes and spleen [83]. Amongst different cell populations, intestinal dendritic cells and macrophages are major migrants from the gut to the CNS after stroke [83]. These results indicate that stroke induces preferential migration of immune cells from the small intestine to the CNS in the acute phase. Moreover, increased trafficking of T cells and neutrophils departing from the small intestine to lymphoid sites such as bone marrow, spleen and lymph nodes (Fig. 4).

In stroke, the pathophysiological role played by the gut-brain axis is exemplified by changes in gut microbiota composition caused by dysphagia, or difficulty in swallowing, a common feature observed in patients [84–86]. Nevertheless, it is evident that the intestinal microbiota composition plays an important role in patient’s outcome as previously summarized by others [87, 88]. Experiments analyzing the use of antibiotics, such as clavulanic acid (AC), in experimental models have shown that alterations in the intestinal flora, induced by these are associated with the reduction of ischemic brain injury [30]. The same authors, have shown that intestinal dysbiosis alters immune homeostasis in the small intestine by inducting bacterial priming of intestinal DCs, leading to the expansion of local T_reg_ cells in the small intestine and suppression of effector IL-17 + γδ T cell function. Right after, these T cells migrate and accumulate in the meninges suggesting directed gut-brain trafficking [30] (Fig. 4).

### Blood

Blood samples have proven particularly valuable for studying the temporal immune response after stroke. Temporal events, such as stroke-induced immune suppression, are well documented to affect patients in the initial days following an ischemic event. Stroke-induced immune suppression involves reduction in lymphocyte counts (lymphopenia) and natural killer (NK) cells in both the blood and spleen [89]. The development of stroke-induced immune suppression has been linked with the development of post-stroke infection and unfavorable functional outcome in patient cohorts [90, 91].

In a similar timeframe after stroke, other cellular dynamics have been identified. An increase in neutrophil number, coupled with lymphopenia, contributes to an elevated neutrophil-to-lymphocyte ratio. Cohort studies showed that a high neutrophil-to-lymphocyte ratio on admission associates with stroke severity and unfavorable functional outcome [92, 93]. At the molecular level, extracellular changes such as high levels of inflammatory factors (e.g., IL-6, C-reactive protein (CRP) and IFN- **γ)** in the serum of stroke patients have been associated with long-term cognitive impairment and unfavorable outcome [94–96].

#### Early Immune Response or Acute Phase

**Fig. 5:**
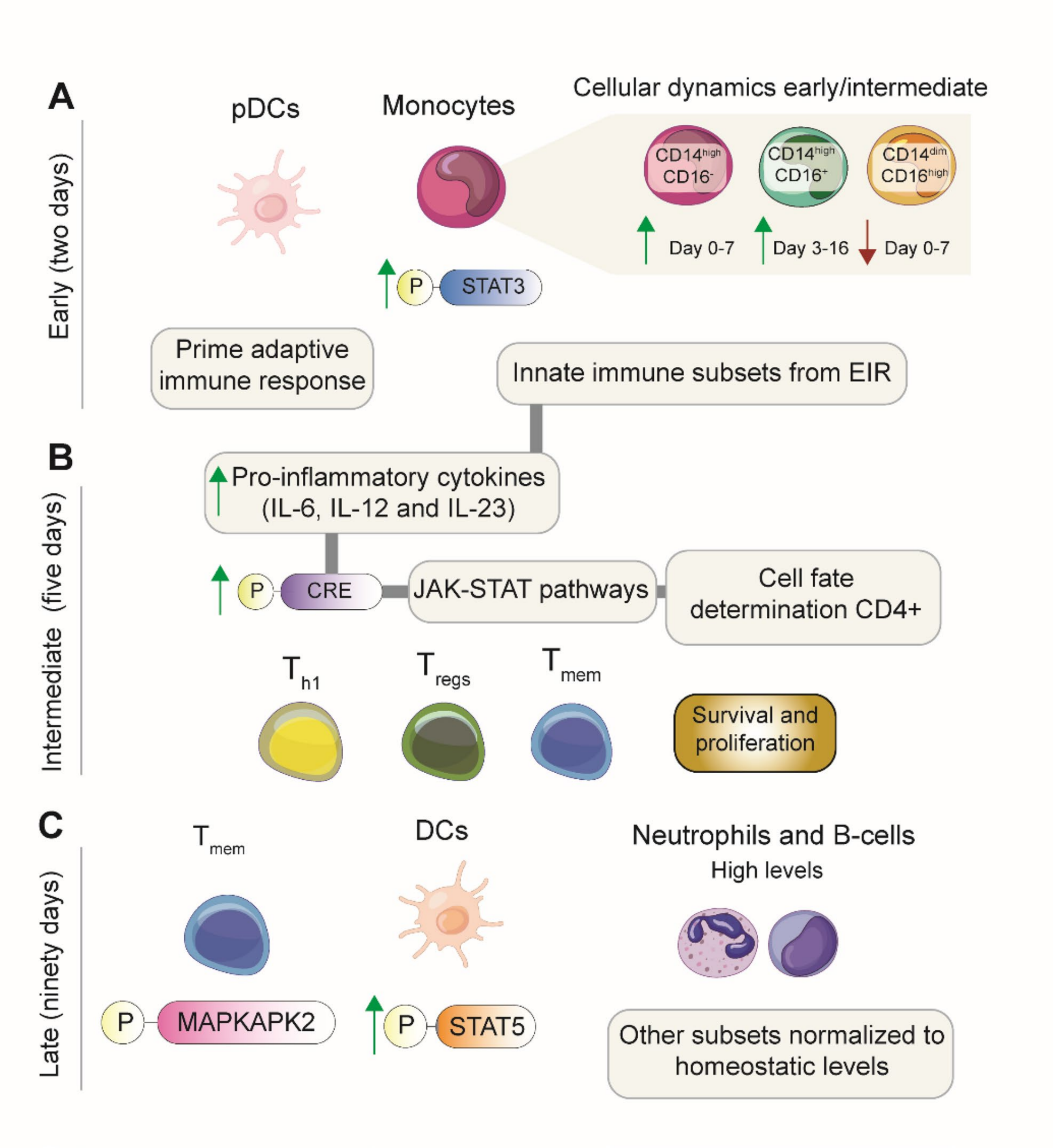
Population dynamics of different immune cell types in peripheral blood of stroke. **A** An initial increase in percentages of plasmacytoid dendritic cells (pDCs) and monocytes is observed. Percentages of classical monocytes (CD14highCD16-) and intermediate monocytes (CD14highCD16+) show an increase from days 0 to 7 and 3 to 16, respectively, whereas percentages of non-classical monocytes (CD14dimCD16high) will decrease from day 0 to 7. **B** Five days after ischemia, the intermediate phase starts, which is characterized by increased levels of T-cell lymphocytes, which persist for several weeks. **C** A late phase is described after 90 days and characterized by elevated levels of neutrophils and B cells in the peripheral blood of stroke patients together with elevated levels of phosphorylated MAPKAPK2 in Th1 and STAT5 in pDCs. Cellular frequencies of other immune subsets are normalized to homeostatic levels

An in-depth approach using immune profiling with mass cytometry (CyTOF) described the evolving immune response in ischemic stroke patients [9]. Their cohort included mostly male patients that underwent acute treatments such as IV-tPA (intravenous tissue plasminogen activator (20.8%), Intra-arterial therapy (IAT, 4.2%) or both (33.3%). During the early immune response, there is an elevation in the phenotypic distribution of immune cells like monocytes and plasmacytoid dendritic cells (pDCs) in the peripheral blood of stroke patients [9]. These cells migrate to the injury sites, in the ischemic tissue, with monocytes differentiating into monocyte-derived macrophages (MDMs) that acquire potent phagocytic properties [97]. Concurrently, DCs, including pDCs, play an important role initiating adaptive immune response by priming of naïve CD8 + T cells [98, 99] and T_regs_ expansion[100], in non-stroke settings (Fig. 5a).

The enrichment of monocyte populations aligns with previous flow cytometry analyses, revealing dynamic changes in classical, intermediate and non-classical monocyte populations. Over time after stroke, CD14^high^CD16- classical monocytes increase between day 0–7 after stroke, while CD14^high^CD16^+^ intermediate monocytes increase between 3 and 16 days, and on the contrary, CD14^dim^CD16^high^ non-classical monocytes decrease from 0 to 7 days post-stroke [101]. However, data reported are not consistent with others reporting no alterations in CD14^high^CD16^+^ monocytes percentages, whereas CD14^high^CD16^+^ monocytes increased and CD14^dim^CD16^+^ monocytes decreased [102] (Fig. 5a). The latter study included both patients with ischemic and hemorrhagic strokes [102]. These differences in between the studies might suggest difference in immunological features between ischemic and hemorrhagic stroke.

At the molecular level, activation of the STAT3 signaling pathway is observed in innate components shortly after stroke, leading to an upregulation of IL-6 in blood [9]. This metabolic stimulus is also witnessed during sterile inflammatory tissue response, e.g., mechanical neurological injury [103]. Elevated phosphorylated STAT3 (pSTAT3) levels in innate immune cells during the acute phase has been associated with poor cognitive recovery after stroke in both animals and patients after stroke [9] (Fig. 5a). Experimental evidence of additional deleterious effects caused by STAT3 includes a hyperactive form of STAT3 induces the development of IL-17-producing helper T (T_H_) cells or pro-inflammatory TH (T_Hi_) in non-stroke settings [104]. Antagonistically, anti-inflammatory regulatory T cells (T_regs_), enriched in later stages of stroke, are shown to rescue deleterious effects of STAT3 hyperactivation in glial cells, such as astrocytes [5].

In blood of patients after ischemic stroke monocytes and DCs are relatively lowly represented cell types compared to other cell types like neutrophils. Traditionally, increased levels of neutrophils have been described as prominent acute peripheral immunological feature following ischemic stroke [32, 34]. In the cohort from Tsai et al., 2019 an increase in neutrophil percentage observed in the early phase after stroke [9]. However, neutrophil enrichment was not pointed as an early immune response factor in their computational modeling. This finding was hampered by individual patient variation over time. Increases in neutrophil levels, on the other hand, were pointed out as characteristic components of the late phase [9].

#### Intermediate Immune Response

As the inflammatory response progresses, neutrophil levels increase quickly after stroke, and factors produced by monocytes and dendritic cells contribute to the expansion of adaptive immune components, particularly T-cell lymphocytes, in the intermediate immune response phase, starting around five days post-stroke [9]. The intermediate phase is characterized by an enrichment of T_regs_ and T_h_1 cells, with a shift towards a pro-inflammatory T_h1_ phenotype associated with poor neurological outcome in experimental models [105] (Fig. 5b).

In contrast to the acute phase, the most enriched components in the peripheral blood of stroke patients are adaptive immune cells, mainly T-cell lymphocytes, such as T_regs_ (CD4^+^CD25^+^FoxP3^+^) and T_h_1 cells (Tbet^+^CD4^+^CD45RA^+^) [9]. T_regs_ are important neuroprotective immune components by diverse mechanisms such as controlling neutrophil deleterious properties [106] or inducing white matter regeneration [8]. T_h_1 cells on the other hand, are important on macrophages activation, and by consequence responsible for cell-mediated immunity and phagocyte-dependent protective responses [107]. Therefore, it might be reasoned that levels of T_h_1 enrichment might be associated with the amount of focal damage.

Analysis of the T_h_1/T_h_2 balance in peripheral blood of ischemic stroke patients in the intermediate phase reveals an enrichment of CD4^+^ T cells adopting a pro-inflammatory T_h_1 phenotype [108–110]. In mice after transient focal ischemia, this imbalance, driven by increased T_h_1 pro-inflammatory response, is associated with poor outcome [105].

Molecular features during the intermediate phase in the model by Tsai et al. include increased cAMP response element-binding protein (CREB) signaling responses in T-cell subsets. Elevated levels of phosphorylated CREB (pCREB) in CD4^+^ subpopulations, such as T_mem_, T_h_1 (Tbet^+^CD4^+^) cells and T_regs_, might be associated with cell cycle regulation of these lymphocytes [9]. CREB is recognized for its role in survival and proliferation of T-cells, including pro-inflammatory (T_h_1-mediated) and anti-inflammatory (T_reg_-mediated) T-cell subsets [111]. Pro-inflammatory cytokines such as IL-6, IL-12, and IL-23, produced by pCREB^+^ cells, promote a proliferation feedback loop and contribute to prolonged survival among the B and T-cell lymphocytes via JAK/STAT signaling pathways, persisting for several weeks [9, 112] (Fig. 5b).

Additionally, the CREB-induced JAK/STAT pathway plays a crucial role in cell fate determination of CD4^+^ cells [113], including the generation and maintenance of T_regs_ [114] (Fig. 5b). After these, at the end of the intermediate phase, numbers of T-cell lymphocytes are thought to normalize towards homeostatic levels before the late response. At this moment, higher levels of B-cell lymphocytes and neutrophils were described to persist at elevated later stages in peripheral blood of stroke patients [9].

#### Late Immune Response

At the cellular level, the late immune response phase, emerging approximately 90 days after the onset of ischemic stroke, is characterized by persistent higher levels of neutrophils and immunoglobulin M^+^ (IgM^+^) B cells [9] (Fig. 5c). While neutrophils are recognized as rapid responders and are linked to detrimental effects their role in the chronic stages remains not fully established. There is speculation that neutrophils might mediate beneficial effects in stroke models involving reperfusion [34]. Consequently, further exploration of differential neutrophil heterogeneity over time during stroke is imperative. Moreover, investigating neutrophil crosstalk with other cells around the ischemic site in the later stages of stroke may unveil potential mechanisms of neutrophil-mediated tissue recovery.

### Targeting (over) Inflammation Via Neuroprotection and Regeneration

To date, only a few attempts have been made to identify immunomodulatory drug candidates demonstrating sufficient efficacy and low systemic toxicity in stroke patients in clinical trials. Among the possible novel investigational treatments that target post-stroke pro-inflammatory cellular or molecular components emerge as a promising candidate. As mentioned in the previous sections, several pro-inflammatory leukocyte populations can increase neuronal damage after stroke. Enlimomab (murine intercellular adhesion molecule-1 (ICAM-1) antibody), a drug that reduces leukocyte adhesion at the injury site in animal models, has been tested in a clinical trial. Although enlimomab has shown moderate positive effects in experimental models [115] it did not improve patient outcome in the tested dosages [116]. In fact, enlimomab significantly worsened stroke patient’s outcome, with predominant infections and fever events being observed in the enlimomab treatment than placebo [116]. Similarly, Natalizumab, a monoclonal antibody against the CD49d receptor that target the α chain of the adhesion molecule very late antigen-4 (VLA-4) was used to target leukocyte migration into the CNS [117]. Although adverse events were not observed, natalizumab administration within 24 h after ischemia did not improve patient outcomes [117].

Considering the proven deleterious pro-inflammatory effects of lymphocyte accumulation in the infarction area after ischemic stroke [118], fingolimod, a drug that prevents the egress of lymphocytes from lymph nodes and limits their recirculation [119] has been clinically tested alone [119] and in combination with a plasminogen activator, alteplase [120]. The first trials show that fingolimod, with or without alteplase, is well tolerated, attenuated reperfusion injury, and improved clinical outcomes in ischemic stroke patients. In an early phase clinical study involving 11 patients after ischemic stroke fingolimod-treated patients showed lower circulating lymphocyte counts, milder neurological deficits, and better recovery of neurological functions [119]. Similar results were observed in a randomized pilot trial involving 22 patients combining fingolimod and alteplase [120]. A meta-analysis including 228 patients showed that fingolimod administered alongside standard treatment increased the likelihood of achieving favorable functional outcomes (modified Rankin Scale [mRS] 0–1 at 90 days; risk ratio [RR] 2.59, 95% CI 1.48–4.56) and was associated with lower NIHSS scores and reduced infarct growth compared with standard treatment alone [121]. Consistent with these findings, a randomized clinical trial reported that coadministration of fingolimod with alteplase in patients treated within a 4.5–6-hour time window was associated with greater early neurological improvement (as reflected by a reduction in NIHSS scores at 24 h) and a favorable shift in functional outcome at 90 days, along with enhanced reperfusion, including both anterograde flow and collateral circulation [122]. While these results are encouraging, larger studies in diverse patient populations are required to confirm efficacy and establish long-term safety.

In this context, pro-inflammatory pathways activated early after stroke have been strongly implicated in secondary brain injury and worse clinical outcomes. For example, elevated levels of interleukin-1 (IL-1) are associated with poorer outcomes in ischemic stroke [123]. In view of this, the Interleukin-1 receptor antagonist protein (rhIL-1ra) has been tested, as an infusion treatment, in two phase 2 randomized trials involving in total 114 ischemic stroke patients [123, 124]. The initial results indicate biological activity of the administered dosage of rhIL-1ra given the reduction in total white cell count, neutrophil count, and plasma CRP and IL-6 concentrations, together with minimal or no disability at 3 months in recipient patients compared with placebo. Initial results, about rhIL-1ra indicated that this inhibitor is safe and well tolerated in patients [123, 124].

While interest in immune checkpoint blockade and other immunomodulatory strategies has grown, particularly in oncology, their application in ischemic stroke remains limited by challenges related to efficiency, safety, and feasibility [125], and they are likely to be most effective as adjuncts to reperfusion therapies. In this context, CAR-T-based immunomodulation may offer a more precise means of controlling excessive inflammatory responses. Building on these concepts of immunomodulation, additional candidate strategies include enhancing T_regs_ proliferation in the cervical lymph nodes to promote anti-inflammatory responses and facilitate tissue repair processes, alongside approaches aimed at modulating tissue-damaging lymphocyte responses, including those mediated by CD8⁺ T cells. Altogether, these strategies may be most effective when timed to the early post-stroke phase following reperfusion, when increased blood–brain barrier permeability facilitates immune cell trafficking into the injured tissue.

### Study Limitations

Although the CNS has traditionally been accepted as an immune-privileged organ due to protection afforded by the BBB and consequent restricted leukocyte access, besides the presumed absence of lymphatic drainage, this view is now considered incomplete. Rather than being immune-isolated, the CNS maintains specialized immune regulation: microglia provide continuous innate surveillance, peripheral immune cells interact with the meninges and choroid plexus, and meningeal lymphatic vessels facilitate antigen drainage to cervical lymph nodes [16, 58]. Thus, immune access is normally tightly controlled, but not absent.

This review showed that this intricate balance is rapidly disrupted after ischemic stroke. Breakdown of the neurovascular unit allows an excessive leukocyte infiltration and exposes CNS antigens to the systemic immune system, physiologically shifting controlled immune surveillance toward dysregulated neuroinflammation. Together, these events contribute to both local neuronal injury and systemic immune dysregulation. Understanding post-stroke pathology therefore requires moving beyond the classical concept of immune privilege toward a dynamic model of bidirectional CNS–immune communication that becomes pathologically amplified after ischemia—particularly following reperfusion. Thus, recapitulating clinical settings in closer proximity.

While both permanent and transient middle cerebral artery occlusion models are widely used, there is a portion of preclinical work focusing on ischemia without recanalization, and thus without robust reperfusion, limiting insights into reperfusion-associated immune response [126, 127]. Although reperfusion-capable models are increasingly implemented, heterogeneity across experimental designs still complicates translational interpretation of immune mechanisms that specifically emerge after successful reperfusion in patients [128, 129]. Importantly, these experimental findings should be interpreted within the context of the typical clinical stroke population [130]. Patients with ischemic stroke are predominantly elderly and frequently present with multimorbidity, including atherosclerosis, diabetes, and cardiovascular disease, conditions known to substantially alter both innate and adaptive immune responses [36, 129]. Age-associated immune remodeling (immunosenescence) and chronic low-grade inflammation (“inflammaging”) may therefore shape post-stroke immune dynamics in patients and represent an important challenge to be experimentally reproduced [131, 132].

While the three-phase model proposed by Tsai and colleagues provides an important framework for understanding the temporal evolution of peripheral immune responses after stroke, it currently focuses primarily on blood-derived immune dynamics [9]. Extending this model to include additional compartments—such as the brain, cervical lymph nodes, spleen, and bone marrow—will be essential for capturing the full spectrum of neuroimmune communication after ischemia. Moreover, the temporal boundaries of the “early phase” are constrained by the limited availability of blood sampling in the first hours after stroke onset. As a result, immune alterations occurring within minutes to the first 24 h are underrepresented, and the current definition of the early phase beginning at ~ 2 days may overlook critical rapid inflammatory events, particularly in patients receiving reperfusion therapies. Incorporating higher-resolution early time points and multi-compartment sampling will therefore help refine this model and increase its translational relevance.

## Stroke-Immunology: the Integrative Approach

This review described cellular and molecular immune events that follow ischemic stroke in a temporal manner in the brain and adjacent areas, lymphoid compartments, the gut, and blood. Yet, a clear-cut image of the temporal immune cascades after ischemic stroke is obscured by disease etiology heterogeneity and preexisting clinical patient conditions [133]. Being able to detect common immune temporal enrichments and their migration patterns is essential for targeted immune modulation. Largely, due to the lack of longer-term time-course studies, more detailed information of these events is restricted to the early phases after stroke.

First, early immune events, including rapid neutrophil infiltration into the brain, are consistently documented in human post-mortem neuropathological studies and recapitulated in experimental models [33, 35]. The ischemic microenvironment shapes the cellular state of myeloid-derived cells and neutrophils during the early and intermediate phases [15]. Adjacent to the brain, the glymphatic system’s basic functions of clearance of harmful components is quickly impaired [55]. Direct communication between the above-cited compartments and the cervical lymph nodes is promoted by the CSF priming the proliferation of pro-inflammatory macrophages and anti-inflammatory T_regs_ followed by migration towards the infarction site [16, 58–60]. Reduction in splenic sizes is observed in patients [66] and experimental models [64] during the transition from the early to intermediate phases. And similarly to the cervical lymph nodes, cellular frequencies of T_regs_ are augmented in the splenic microenvironment [67]. Concomitant to these facts, myeloid lineage commitment in hematopoietic progenitors will be observed in the bone marrow. This event may impair B-cell lymphopoiesis and is consistent with the peripheral lymphopenia commonly observed in patients with ischemic stroke [74]. Moreover, early events such as the enrichment of monocytes and plasmacytoid dendritic cells (pDCs) and activation of the STAT3 signalling pathway in innate immune cells are reported to occur in the blood of stroke patients [9]. Overall, early immune events include the quick proliferation of innate immune pro-inflammatory components that might help with the removal of dead cells and cellular debris, besides triggering the proliferation of adaptive immune cells, mainly T-cell lymphocytes, benchmarking the intermediate phase. In the ischemic brain, these T-cell lymphocytes migrate to the lesion’s periphery and gradually accumulate within the infarct region over time, playing both pro- and anti-inflammatory roles [134]. Highly divergent from homeostatic peripheral levels, the ratio of T_h_1/T_regs_ is 1:1 in the ischemic brain [5]. Little is known about intermediate immune response in compartments other than the brain and blood. The late immune response is characterized by high levels of neutrophils and immunoglobulin M+ (IgM+) B cells in the blood of patients [9]. B cells, similar to T_regs_, are reported to promote neurodegeneration in stroke settings [6].

While the negative impact of exacerbated pro-inflammatory response is well witnessed in ischemic settings, natural neuroregenerative processes are only recently uncovered. More specifically, molecular mechanisms regarding T- cell lymphocytes in the ischemic brain have gained attention over the last years, given the breakthrough in identifying T_regs_ [5, 7, 8] as a major anti-inflammatory and regenerative player after ischemic stroke. The robustness of the data regarding the neuroprotective role of T_regs_ may gain interest within the cellular therapy-based approaches. Despite these benchmark findings, knowledge gaps remain elusive, such as the cervical lymph nodes as an enrichment site for T_regs_ in patient material or cellular phenotype of brain-infiltrated neuroprotective T_regs_. This information could lead to the design of immunomodulatory approaches targeting specific cellular enrichment in the cervical lymph nodes or other sites.

Later regenerative mechanisms such as B-cell mediated tissue regeneration remain largely unexplored. To date, the exact migration routes of diverse immune components and the connection between these main events have not been clarified yet. Detecting common immune temporal enrichments and their migration patterns is essential for targeted immune modulation. A deeper look at cellular states using single-cell genomics approaches such as scRNA-seq or spatial transcriptomics is essential for connecting temporal events using trajectory analysis. This approach may evidence new layers of information regarding different cellular states and how those can correlate with patients’ outcomes and could improve drug intervention accuracy.

Eventually, complementary therapies targeting inflammation based on cellular states rather than cell types might ameliorate excessive neuronal damage often observed in stroke pathology. Later on, artificial manipulation to reduce pro-inflammatory-caused neuronal death, combined with neuroregeneration-based approaches, such as cellular enrichment of T_regs_, may pave the way for new therapeutic avenues after stroke. Overall, this narrative review provides temporal evidence that could open a new route towards designing innovative immunomodulation-based treatments for ischemic stroke patients.

## Data Availability

The figure files presented in this Review are available from the corresponding author upon reasonable request.

## Abbreviations

BBB: Blood–brain barrier
BDNF: Brain–derived neurotrophic factor
bDC: Brain dendritic cells
cAMP: Cyclic adenosine monophosphate
CI: confidence interval
CNS: Central nervous system
CREB: cAMP response element–binding protein
CRP: C–reactive protein
CyTOF: Cytometry by time–of–flight (mass cytometry)
DAMPs: Damage–associated molecular patterns
DC: Dendritic cells
ICAM: 1–Intercellular adhesion molecule–1
IL: Interleukin
MMPs: Matrix metalloproteinases
mRS: Modified Rankin Scale
NETs: Neutrophil extracellular traps
NGF: Nerve growth factor
NIHSS: National Institutes of Health Stroke Scale
NK: Natural killer cells
rhIL: Recombinant human interleukin
RR: Risk ratio (relative risk)
T_h1_: T helper 1 cells
T_regs_: Regulatory T cells
VLA-4: Very late antigen-4
